# Adrenalectomy for solitary recurrent hepatocellular carcinoma five years after living donor liver transplantation: A case report

**DOI:** 10.1016/j.ijscr.2018.11.062

**Published:** 2018-11-27

**Authors:** Mohamed Abdel Wahab, Ahmed Shehta, Eman M. Ibrahim, Rehab T. Eldesoky, Ahmed A. Sultan, Khaled R. Zalata, Omar Fathy, Mohamed Elshoubary, Tarek Salah, Amr M. Yassen, Mohamed Elmorshedi, Ahmed Monier, Ahmed Farouk, Usama Shiha

**Affiliations:** a Department of Surgery, Gastrointestinal Surgery Center, College of Medicine, Mansoura University, Egypt; b Department of Pathology, College of Medicine, Mansoura University, Egypt; c Department of Anesthesia and Intensive Care, Gastrointestinal Surgery Center, College of Medicine, Mansoura University, Egypt; d Diagnostic & Interventional Radiology Department, Gastrointestinal Surgery Center, College of Medicine, Mansoura University, Egypt

**Keywords:** HCC, hepatocellular carcinoma, HCV, hepatitis C virus, LDLT, living-donor liver transplantation, CT, computed tomography, MELD, model for end stage liver disease, GRWR, graft to recipient weight ratio, US, ultrasound, SVR, sustainedvirologic response, Living donor liver transplantation, Hepatocellular carcinoma recurrence, Adrenalectomy, Direct-acting antiviral agents

## Abstract

**Introduction:**

The adrenal gland is a rare site for hepatocellular carcinoma (HCC) recurrence after living-donor liver transplantation (LDLT). Solitary adrenal recurrence can be managed by surgical excision, with expected better survival outcomes.

We describe a rare case of successful left adrenalectomy of solitary recurrent HCC in the left adrenal gland 5 years after LDLT.

**Presentation:**

59 years male patient with HCC complicating chronic HCV infection received a right hemi-liver graft from his son. The actual graft weight was 1208 g and GRWR was 1.5. The patient started oral direct acting antiviral drugs for recurrent HCV 2 years after LDLT.

A left adrenal mass was detected on follow up radiology. No other metastatic lesions were detected on metastatic workup. Left adrenalectomy was done by an anterior approach.

The postoperative course was uneventful and was discharged a week after operation. Postoperative pathological and immune-histochemical examinations confirmed the metastatic HCC nature of the mass. The patient is under regular follow up with no recurrences 6 month after resection.

**Discussion:**

There is no consensus regarding the management of HCC recurrence after LDLT. Most patients had multi-organ recurrences and usually offered palliative or supportive care. Solitary HCC recurrence offers a better chance for more aggressive therapy, offering better prognosis.

**Conclusion:**

Solitary adrenal recurrence of HCC after LDLT is extremely rare. Strict follow up protocol is necessary to allow early detection of tumor recurrence. Curative surgical resection is a safe option associated with low morbidity and expected to have a good long-term survival.

## Highlights

• Solitary adrenal recurrence of HCC after LDLT is extremely rare.
• Strict follow up protocol is necessary to allow early detection of tumor recurrence.
• Curative surgical resection of solitary recurrent HCC is a safe option.
• It is associated with low morbidity and expected to have a good long-term survival.

## 1 Introduction

Hepatocellular carcinoma (HCC) is the fourth most common cancer worldwide, and the most common primary hepatic malignancy. Worldwide, the annual incidence of HCC continues to rise owing to the increase of hepatitis C virus (HCV) epidemic, obesity and nonalcoholic steatohepatitis [1].

Liver transplantation remains the main therapeutic option for selected HCC patients. It has the advantage of removing not only the primary hepatic tumor but also the background of liver cirrhosis [2]. Liver transplantation patients could achieve better disease free and overall survival in comparison with other treatment modalities [3,4]. However, tumor recurrence remains a major problem after liver transplantation. It is estimated that about 10–20% of HCC patients will experience tumor recurrence after liver transplantation [[5], [6], [7]].

Extrahepatic recurrence of HCC is not so common after liver transplantation. Most of those patients experience multi-site recurrences and usually offered palliative or supportive care. The prognosis of such patients is usually poor [8]. On the other hand, solitary HCC recurrence offers a better chance for more aggressive therapy, offering better prognosis [9].

The adrenal gland is a rare site for HCC recurrence, especially after liver transplantation. Solitary adrenal HCC recurrence can be managed by surgical excision, with expected better survival outcomes. Very few reports had addressed solitary adrenal recurrence of HCC that was successfully managed with surgical excision [10,11].

In this report, we describe a rare case of successful left adrenalectomy of solitary recurrent HCC in the left adrenal gland 5 years after living-donor liver transplantation (LDLT). This work has been reported in line with the SCARE criteria [12].

## 2 Case presentation

**Fig. 1:**
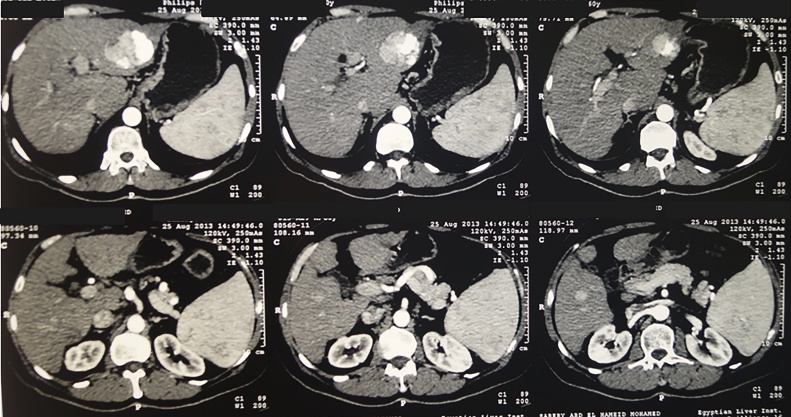
Abdominal computed tomography before liver transplantation showing large left hemi-liver hepatocellular carcinoma with partial lipidol uptake and other smaller ones in both hemi-livers.

A 59 years old male patient with HCC complicating liver cirrhosis due to chronic HCV infection, was planned for LDLT. He had a history of three sets of trans-arterial chemo-embolization. Preoperative triphasic abdominal computed tomography (CT) showed enlarged cirrhotic liver with large left hemi-liver HCC 4.8 * 5.8 cm with partial lipidol uptake with residual viable tumor tissue, and other smaller HCCs in both hemi-livers with no lipidol uptake, and mild enlarged spleen (Fig. 1). His preoperative Child-Pugh score was 6 (class A), model for end stage liver disease (MELD) was 9, and alpha feto-protein was 14.1 ng/ml.

He received a right hemi-liver graft without the middle hepatic vein from his son (22 years old). The actual graft weight was 1208 g and graft weight to recipient weight ratio (GRWR) was 1.5.

The surgical technique had been described previously [13]. The graft had double hepatic venous anastomoses. Right hepatic vein (30 mm) and was anastomosed to the recipient right hepatic vein with venoplasty (30 mm). Segment VIII vein (10 mm) was anastomosed to middle hepatic vein stoma using a synthetic vascular graft (polytetrafluoroethylene). The recipient main portal vein stump (20 mm) was anastomosed, in end to end fashion, to the graft portal vein (13 mm). Then arterial reconstruction was done between the graft right hepatic artery (3 mm) and the recipient left hepatic artery (3 mm). Doppler ultrasound (US) was performed upon completion of all vascular anastomoses and showed sound anastomoses and adequate inflow and outflow of the graft without congestion.

Double biliary anastomoses were performed duct-to-duct technique over 2 trans-anastomotic biliary catheters (4 french) exiting through a separate opening into the common bile duct. The graft right posterior sectorial duct (3 mm) was anastomosed to the recipient common hepatic duct (4 mm), and the graft right anterior sectorial duct (3 mm) was anastomosed to the recipient cystic duct (3 mm). Completion cholangiogram showed adequate biliary anastomoses with no leakage.

Postoperative pathological examination of the explanted liver showed cirrhotic liver with well differentiated HCC (left hemi-liver lesion: 6 cm & right hemi-liver lesion: 1 cm) with occasional vascular tumor emboli.

The patient developed early hepatic artery thrombosis on the first postoperative day that was managed by angio-intervention. Afterwards, the patient had smooth postoperative course and was discharged 3 weeks after the operation. The patient received our regular immunosuppression protocol and maintained on Cyclosporine [13]. He was planned to regular follow up in outpatient visits including detailed laboratory and radiological evaluation.

The patient started oral direct acting antiviral drugs for recurrent HCV 2 years after LDLT. The patient received Ombitasvir (12.5 mg), paritaprevir (75 mg), and ritonavir (50 mg) plus Ribavirin in August 2015 for 3 months, but relapse occurred after 6 months. Then he received Sofosbuvir (400 mg) plus Daclatasvir (60 mg) plus Ribavirin in October 2016 for 6 months, but also relapse occurred again. Finally, he received Sofosbuvir (400 mg) plus Simeprevir (150 mg) plus Ribavirin in April 2017 for 6 months but relapse occurred after 1 month of completion of the therapy.

**Fig. 2:**
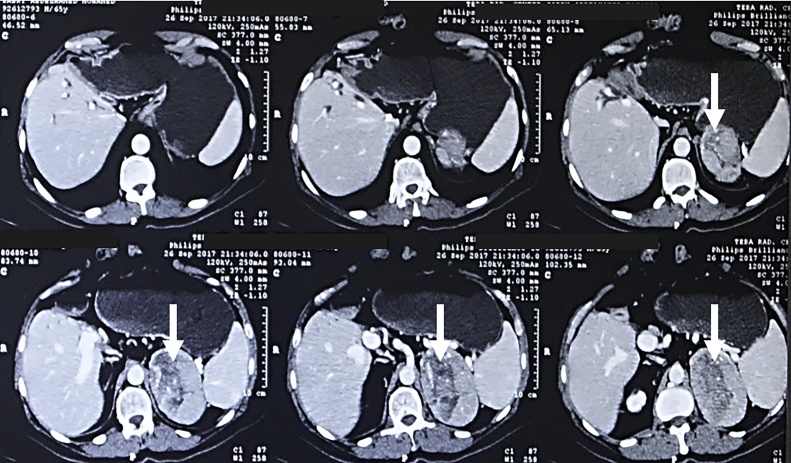
Abdominal computed tomography 5 years after liver transplantation showing large left adrenal mass (white arrow).

On follow up abdominal US, a left adrenal mass was detected. Triphasic CT abdomen showed normal right hemi-liver graft and its vasculature and left adrenal mass 9 cm in size suggesting metastatic HCC (Fig. 2). Further metastatic workup was performed including CT chest, and bone scan and no other metastatic lesions were detected. Serum alpha feto-protein was 85.4 ng/ml, and 24-hour urinary valinyl mandelic acid was normal (3 mg/24 h). The decision was to proceed for surgical resection.

Left adrenalectomy was done by an anterior approach. Anterior approach was preferred to allow for adequate evaluation of the abdominal cavity and better manipulation of large tumor. The spleen, pancreas and the left colon were moved aside to expose the mass. The adrenal vessels were identified and divided, and the mass was removed. The patient had smooth postoperative course and was discharged 7 days after the operation.

**Fig. 3:**
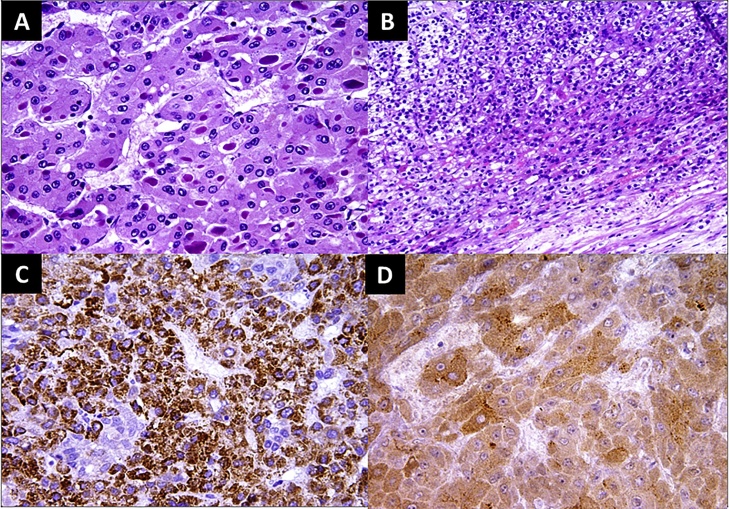
Postoperative photomicrographs: (A) The neoplastic cells are arranged in thick trabeculae and nests surrounded by sinusoidal vessels. Intracellular eosinophilic hyaline bodies are frequent (Hx&E – ×400). (B) Compressed adrenal gland at the periphery of the tumor (Hx&E – ×200). (C) Immunohistochemistry for Hep Par-1 revealed granular cytoplasmic staining of the neoplastic cells (×400). (D) Immunohistochemistry for Glypican-3 revealed granular cytoplasmic staining of the neoplastic cells (×400).

Postoperative pathology showed a single well circumscribed firm mass 13 × 9 cm in size. It was greyish white in color with areas of hemorrhage and necrosis. Microscopically, the tumor is formed of sheets of atypical polygonal cells with evident sinusoidal pattern. The tumor cells exhibit moderate degree of anaplasia (Fig. 3). Immunohistochemical study showed focal positivity for Hep Par-1 and Glypican-3, while negative for chromogranin, synaptophysin and s-100. Metastatic HCC was confirmed (Fig. 3).

**Fig. 4:**
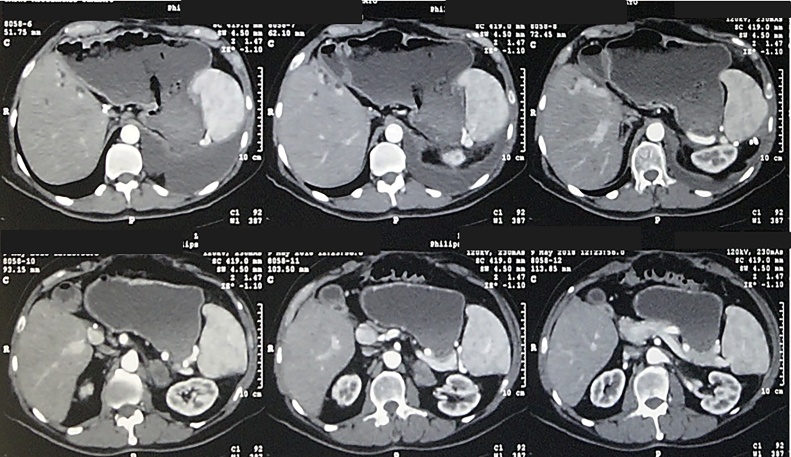
Abdominal computed tomography 6 month after excision of metastatic adrenal hepatocellular carcinoma.

The patient is under regular follow up visits in the outpatient clinic and no evidence of new tumor recurrences for 6 months after excision (Fig. 4).

## 3 Discussion

HCC recurrence represents a serious problem after liver transplantation as it is directly associated with recipient mortality. In spite of the proper selection of early HCC patients for liver transplantation, HCC recurrence may occur. It is reported that about 10–20% of liver transplant recipients will experience HCC recurrence [[5], [6], [7],^14^].

Recurrence may occur in the implanted liver graft or in extrahepatic sites. Extrahepatic recurrence commonly occurs in the portal vein, peritoneal cavity, lymph nodes, bone, and lungs [15]. In a large series of liver transplantation for HCC, Barry et al. reported tumor recurrence in 117 patients (13.5%). The most common sites for recurrence were lungs (59%), abdominal cavity (38%), and the liver (35%) [16]. Those patients usually have multi-site recurrences and can only be offered palliative therapies or only symptomatic treatment [8].

The adrenal gland is a rare site for HCC recurrence. Solitary adrenal recurrence of HCC after liver transplantation is limited to few reports [10,11,17]. Such recurrence pattern offers a unique opportunity for those patients to be managed by surgical resection, with expected better survival outcomes. In our case, solitary HCC recurrence occurred in the left adrenal gland 5 years after LDLT. This could be successfully managed by left adrenalectomy. The postoperative course was uneventful and has no recurrences 6 month after left adrenalectomy.

The mechanism of extrahepatic recurrence of HCC, especially after liver transplantation is unclear. It had been postulated that it may be due to the growth of occult metastases before transplantation or the engraftment of circulating tumor cells that passes into the systemic circulation during the operation [18].

In the recent years, direct-acting antiviral drugs have been introduced as a new hope for HCV management. Initial reports regarding those novel medications have achieved a dramatic improvement of sustained virologic response (SVR) rate reaching more than 90% of the HCV treated patients [[19], [20], [21]]. So, it was assumed that, HCC occurrence and recurrence will continue to decrease with higher SVR rates with direct-acting antiviral drugs.

On the contrast, recent reports regarding this issue reported unexpectedly high rates of denovo HCC and HCC recurrence [[22], [23], [24]]. The results of those reports are quite conflicting and did not reach a solid evidence regarding the safety of those agents, and the appropriate candidates for those agents.

One of the great concerns in our report is the repeated regimens of direct-acting antiviral drugs for HCV recurrence after transplantation. The patient had a highly-resistant form of HCV that failed to achieve a satisfactory SVR with different regimens. The relationship between direct-acting antiviral agents and HCC occurrence or recurrence and the underlying possible mechanisms remain controversial and there is no enough evidence to prove or disprove this relationship.

Currently, there is no consensus regarding the management of HCC recurrence after liver transplantation. Most of those patients had recurrences in multiple organs and usually offered just palliative or supportive care. Treatment of those patients is not always effective, making their prognosis poor [8]. Different therapeutic modalities had been proposed as surgical excision, chemo-embolization, radiotherapy, and alcohol injection [9,25]. However, the treatment modalities are tailored according to each case.

On the other hand, solitary HCC recurrence offers a better chance for more aggressive therapy, offering better prognosis and outcome [9]. In our case, solitary HCC recurrence occurred in the left adrenal gland, which could be successfully managed by left adrenalectomy. The postoperative course was uneventful and has no recurrences 6 month after resection. However, more long-term follow up is needed to prove the efficacy of the management.

Another important point that should be addressed in the management of recurrent HCC after liver transplantation is the early detection of recurrent HCC. Early detection of recurrent HCC allows for more aggressive therapy, which potentially improves the patient’s survival. Patient compliance to strict follow up program is mandatory for early detection of tumor recurrence allowing early intervention. Follow up protocols should include evaluation of both liver graft and extrahepatic sites. Scortegagna et al. recommended to add extrahepatic surveillance to the follow-up protocol after liver transplantation and maintain a low threshold for cross-sectional imaging in symptomatic patients [1]. In our case, early detection of solitary recurrent HCC allowed early intervention, which is associated with better outcomes.

In conclusion, solitary adrenal recurrence of HCC after LDLT is extremely rare. Strict follow up protocol is necessary to allow early detection of tumor recurrence. Curative surgical resection of solitary recurrent HCC is a safe option which is associated with low morbidity and expected to have a good long-term survival.

## Conflicts of interest

All authors declared that there are no conflicts of interest. This work has been reported in line with the scare criteria

## Funding

No external funding resources.

## Ethical approval

This case report was accepted by the local ethical committee.

## Consent

We obtained a written informed consent from the patient for the publication of this case report and accompanying images. A copy of this written consent is available for review by the editor-in-chief of the international journal of surgery case reports on request.

## Author contribution

Conception and design of the study: Shehta A, Wahab MA.

Collection and assembly of the data: Shehta A, Ibrahim EM.

Data analysis and interpretation: Shehta A, Ibrahim EM.

Drafting the manuscript: All authors.

Critical revision of the manuscript for important intellectual content: All authors.

Final approval of the manuscript: All authors.

Study supervision: Wahab MA.

## Registration of research studies

None.

## Guarantor

Mohamed Abdel Wahab (study supervisor).

Ahmed Shehta (corresponding author).

## Provenance and peer review

Not commissioned, externally peer reviewed.
